# Delay discounting is associated with the fractional amplitude of low-frequency fluctuations and resting-state functional connectivity in late adolescence

**DOI:** 10.1038/s41598-017-11109-z

**Published:** 2017-08-31

**Authors:** Song Wang, Ming Zhou, Taolin Chen, Xun Yang, Guangxiang Chen, Qiyong Gong

**Affiliations:** 1 Huaxi MR Research Center (HMRRC), Department of Radiology, West China Hospital of Sichuan University, Chengdu, 610041 China; 2 Department of Psychoradiology, Chengdu Mental Health Center, Chengdu, 610031 China; 3 School of Sociality and Psychology, Southwest University for Nationalities, Chengdu, 610041 China; 4 Department of Psychology, School of Public Administration, Sichuan University, Chengdu, 610065 China

## Abstract

As a component of self-regulation, delay discounting (DD) refers to an individual’s tendency to prefer smaller-but-sooner rewards over larger-but-later rewards and plays an essential role in many aspects of human behavior. Although numerous studies have examined the neural underpinnings of DD in adults, there are far fewer studies focusing on the neurobiological correlates underlying DD in adolescents. Here, we investigated the associations between individual differences in DD and the fractional amplitude of low-frequency fluctuations (fALFF) and resting-state functional connectivity (RSFC) in 228 high school students using resting-state functional magnetic resonance imaging (RS-fMRI). At the regional level, we found an association between higher DD and greater fALFF in the dorsal anterior cingulate cortex (dACC), which is involved in conflict monitoring and strategy adaptation. At the connectivity level, DD was positively correlated with the RSFC between the dACC and the left dorsolateral prefrontal cortex (DLPFC), a critical functional circuit in the cognitive control network. Furthermore, these effects persisted even after adjusting for the influences of general intelligence and trait impulsivity. Overall, this study reveals the fALFF and RSFC as the functional brain basis of DD in late adolescents, aiding to strengthen and corroborate our understanding of the neural underpinnings of DD.

## Introduction

Self-regulation is a crucial human capacity that allows individuals to inhibit desires, control thoughts, regulate emotions and behaviors, and make choices and plans^1, 2^. One method of evaluating self-regulation is by measuring delay discounting (DD), which refers to an individual’s preference for smaller-but-sooner rewards over larger-but-later rewards^3, 4^. Evidence from many previous studies has suggested that DD is critical for an individual’s mental health and behavioral performance. For example, DD is found to be associated with many mental disorders and problematic behaviors, such as attention-deficit/hyperactivity disorder, obsessive-compulsive personality disorder, anorexia nervosa, pathological gambling and drug use^5–8^. Moreover, DD is associated with many cognitive functions including intelligence, working memory and academic level^9–13^. In summary, DD may play an essential role in an individual’s developmental outcomes.

Over the last decade, a large body of neuroimaging studies have begun to examine the relationships between DD and brain function and structure. Evidence from numerous task-based functional magnetic resonance imaging (fMRI) studies has indicated that DD is primarily determined by three functional brain networks: the cognitive control network, including the anterior cingulated cortex (ACC), the dorsolateral prefrontal cortex (DLPFC) and the ventrolateral prefrontal cortex (VLPFC); the valuation network, including the substantia nigra (SN), the ventral tegmental area (VTA), the ventral striatum (VS), the posterior cingulate cortex (PCC) and the ventromedial prefrontal cortex (VMPFC); and the prospection network, including the amygdala and the hippocampus^14, 15^. Furthermore, several structural MRI studies have reported that individual differences in DD are mainly associated with the cortical thickness or regional gray matter volume in the prefrontal cortex (PFC), including the DLPFC, the VLPFC, the ACC, the medial PFC (MPFC)^16–19^, and the striatum, comprising the caudate and the putamen^17, 20^. Notably, compared to most studies focusing on the neural mechanisms of DD using a task-based fMRI design, relatively few studies have examined the neural correlates underlying DD using task-free designs. Given that DD is generally considered a stable personality trait that varies among individuals^3, 4, 21^, the neural basis underlying DD might be implicated in the overall brain function and structure under task-free conditions.

Resting-state fMRI (RS-fMRI) is one of the tools used to examine the brain bases of personality traits and behavioral performance under task-free conditions^22–24^. This imaging technique can detect resting-state brain activity (e.g., low-frequency fluctuations)^25^, which is reliably related to brain activity during the performance of tasks^26^. The fractional amplitude of low-frequency fluctuations (fALFF) is a widely used method to assess low-frequency fluctuations and reflects the regional properties of intrinsic brain dynamics^27, 28^. Many previous studies have demonstrated that fALFF is behaviorally relevant and can reliably predict human personality traits^29–31^. Resting-state functional connectivity (RSFC) is another popular and reliable indicator reflecting synchronization among multiple brain regions^22, 24, 32^. Evidence from many previous investigations has indicated that RSFC is an excellent predictor of personality traits^33–35^. Thus, fALFF and RSFC are useful tools for uncovering the neural substrates of DD.

In recent years, several studies have employed RS-fMRI to investigate the neural correlates underlying DD^36–41^. However, these studies had several limitations that may limit the statistical power of their findings. First, each of these investigations focused exclusively on RSFC, and most of the RSFC analyses were based on multiple regions of interest (ROIs) [e.g., dorsal ACC (dACC)^36^, fronto-insular cortex^37^, striatum^40^, VMPFC^41^], which were selected from previous studies but not studied in a single group of participants. According to our knowledge, no study has reported the association between DD and regional spontaneous brain activity (e.g., fALFF). Second, the sample sizes were much too small, ranging from 14 to 38 participants, with the exception of two studies (74 participants, Guo and Feng, 2015^41^; 123 participants, Han *et al*. 2013^37^). To acquire adequate statistical power, at least 150 participants are recommended for examining the neural correlates of individual differences in personality and behaviors^42^. Third, the sample characteristics were highly heterogeneous (e.g., disproportionate sex ratio and broad age range). In addition, the participants in each of these studies were adults. There are relatively fewer studies exploring the brain basis underlying DD in young participants (e.g., adolescents). Given that adolescents are characterized by heightened risk-taking and impulsive behaviors, and their brains are undergoing structural and functional reconfigurations^43, 44^, it would be interesting and valuable to explore the neural bases of DD in adolescents.

To our knowledge, no study has used RS-fMRI to investigate the association between DD and intrinsic brain activity in adolescents. However, there are several task-based fMRI studies focusing on the neural basis of DD in adolescents. For example, evidence from a study in 50 healthy participants between ages of 8 and 25 years has showed that delayed behaviors are associated with higher activation in the right DLPFC, right VLPFC, right parietal cortex and left cerebellum, and increased negative functional connectivity between the medial striatum and right DLPFC^45^. Moreover, another investigation based on 40 male healthy participants (age range: 11.96–31.77 years) reported that delayed behaviors were linked with increased activation in the left DLPFC, left temporal and parietal cortex, left precentral gyrus, bilateral cerebellum and bilateral occipital cortex, and decreased activation in the bilateral thalamus, right dorsal striatum and right inferior frontal gyrus^46^. In addition, in 30 adolescents with substance abuse problems (age range: 12–18 years), Stanger *et al*. (2013) found that compared to the immediate choices, delayed choices were associated with lower activation in the right occipital cortex and lingual gyrus^47^. Considering that RS-fMRI is a reliable tool for detecting intrinsic brain activity at rest and is different from task-based fMRI^22, 24^, it is necessary to use RS-fMRI to explore the functional brain basis of DD in adolescents and compare the results with the findings from previous task-based fMRI studies. Given that no study has reported the association between DD and regional spontaneous brain activity, the first aim of the present study was exploring the relationship between DD and fALFF in a sample of high school students. Furthermore, we used the seed-based RSFC analysis to examine the association between DD and RSFC, which is the second aim of this study.

To address these issues, we first used a standard measurement to assess individual differences in DD. Then, we correlated the DD scores with whole-brain voxel-wise fALFF to identify the brain areas related to DD. Considering previously reported functional and structural findings regarding DD in the brain, we hypothesized that the spontaneous brain activity in PFC regions (e.g., the DLPFC, the VLPFC, the MPFC and the ACC) and the striatum might predict individual differences in DD. Next, we used the cluster(s) identified from the fALFF-behavior correlation analyses to calculate the RSFC across the whole-brain and explored the relationship between RSFC and DD. In light of the relation of DD with functional connectivity obtained in previous fMRI and RS-fMRI studies, we further hypothesized that DD might be associated with RSFC in the cognitive control network, valuation network and prospection network. To ensure adequate statistical power for the whole-brain analyses, we investigated the associations between DD and fALFF and RSFC in a large sample of late adolescents (N = 228) with a narrow age range. Finally, we assessed the specificity of the associations between DD and fALFF and RSFC by excluding confounding variables, including general intelligence and trait impulsivity.

## Results

### Identification of the neural basis underlying DD

Table 1 details the descriptive statistics for age, DD, general intelligence and trait impulsivity. According to the conventions^48^, the scores of each behavioral measure may be normally distributed, with skewness and kurtosis values ranging from -0.46 to 0.32. To test whether the scores of behavioral measures were normally distributed, we performed one-sample Kolmogorov-Smirnov test (K-S)^49, 50^. The results showed that all scores were normally distributed [for DD (*lnk*): K-S = 1.33, *p* = 0.060; for general intelligence: K-S = 1.33, *p* = 0.102; for trait impulsivity: K-S = 0.82, *p* = 0.515]. Moreover, DD was not associated with age [r = -0.01, *p* = 0.830 (uncorrected), *p* = 5.81 (Bonferroni corrected)]. There were no gender differences in DD [t (226) = 0.53, *p* = 0.594 (uncorrected), *p* = 4.158 (Bonferroni corrected)]. Next, we explored the neural substrates underlying DD.

**Table 1:** Descriptive statistics for measures (N = 228).

Variable	Mean	SD	Minimum	Maximum	Skewness	Kurtosis
Age	18.48	0.55	16.76	20.44	0.52	1.76
Delay discounting (*lnk*)	−4.24	1.32	−8.75	−1.39	−0.46	0.32
General intelligence	24.22	5.70	6.00	36.00	−0.27	−0.09
Trait impulsivity	63.82	8.58	44.00	86.00	0.14	−0.43

*Note:* N = number; SD = standard deviation.

**Figure 1:**
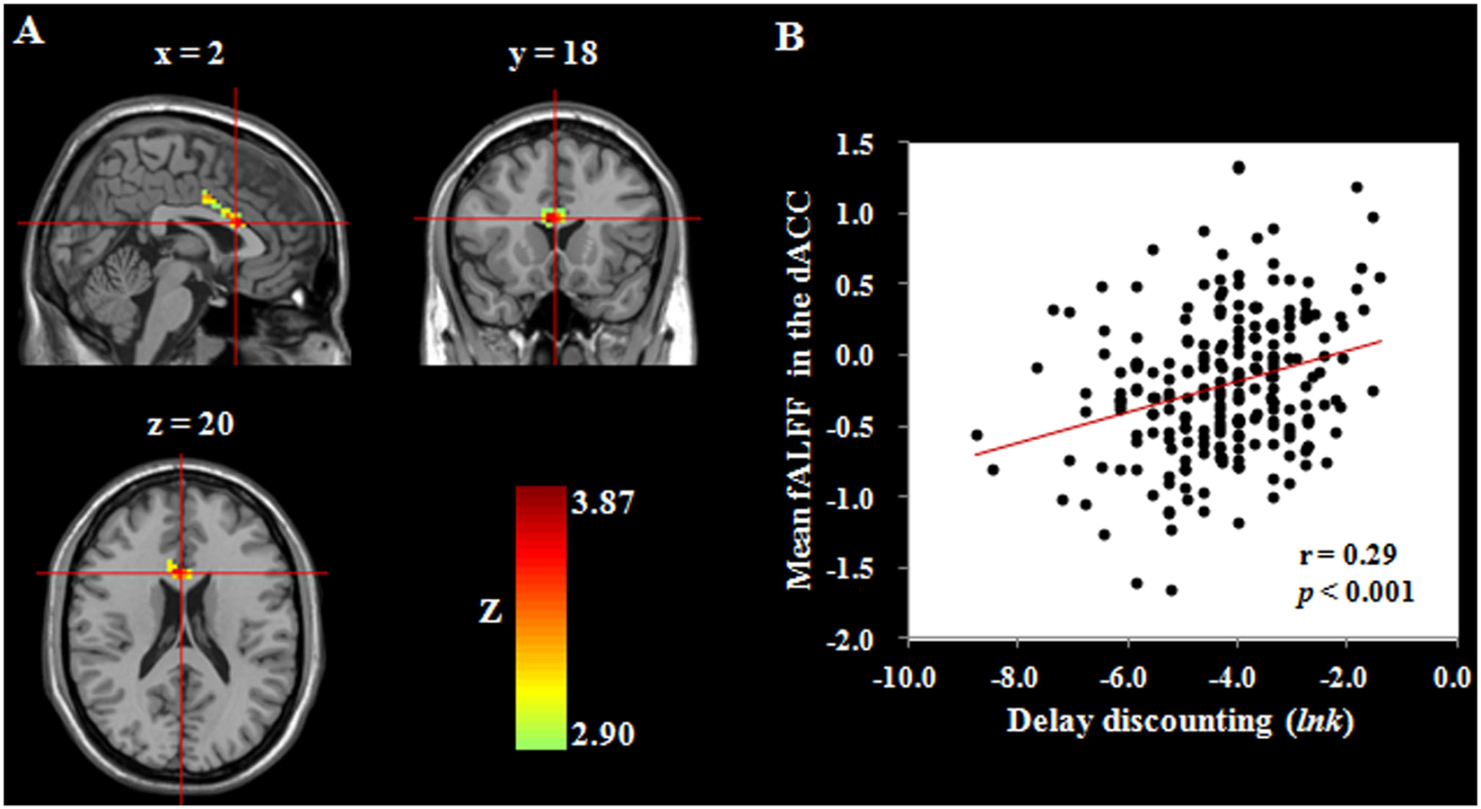
Brain regions related to delay discounting. (**A**) Brain images depicting the positive association between delay discounting and the fALFF in the dACC. (**B**) Scatter plot showing the correlation between delay discounting and the fALFF in the dACC (r = 0.29, *p* = 0.00001). dACC, dorsal anterior cingulate cortex; fALFF, fractional amplitude of low-frequency fluctuations.

First, to identify the brain regions related to DD, we carried out whole-brain correlation analyses between voxel-wise fALFF values and DD scores, controlling for gender, age and head motion (i.e., framewise displacement, FD)^51^. We found a significant association between higher DD and greater fALFF in the dACC (r = 0.29, *p* = 0.00001; see Fig. 1 and Table 2), after correcting for multiple comparisons (Monte Carlo simulation). No other significant results were obtained in these analyses. To evaluate the stability of the relationship between DD and spontaneous brain activity, we performed prediction analyses using the linear regression and four-fold balanced cross-validation procedure. The fALFF in the dACC significantly predicted individual differences in DD [r_(predicted, observed)_ = 0.25, *p* = 0.0001], even after adjusting for gender, age and FD.

**Table 2:** Brain regions that exhibited associations between delay discounting and fALFF and RSFC.

Region	BA	Peak MNI coordinate			Peak Z-score	Cluster size (mm^3^)
		x	y	z		
Correlation with fALFF						
dACC	24	2	18	20	3.87	2754
Correlation with RSFC (dACC as seed)						
Left DLPFC	10	−33	46	12	4.02	3969

*Note:* The significant threshold in each region was set as follows: *p* < 0.05 at the cluster level and *p < *0.005 at the voxel level, at least 50 voxels (1350 mm^3^). MNI = Montreal Neurological Institute; fALFF, fractional amplitude of low-frequency fluctuations; dACC, dorsal anterior cingulate cortex; RSFC, resting-state functional connectivity; DLPFC, dorsolateral prefrontal cortex.

Previous studies have showed that the nuisance regressors (e.g., head motion and non-gray matter signals) in the preprocessing of RS-fMRI data may affect the fALFF in the brain^52^. Thus, we reprocessed our data without the step of nuisance regression and retested the correlation between DD and fALFF. The results revealed that after correcting for multiple comparisons (Monte Carlo simulation), the correlation of DD with the fALFF in the dACC remained (BA24; MNI coordinates: 0, 18, 18; Z = 3.68; r = 0.27, *p* = 0.00005; cluster size = 2,241 mm^3^). We found no other significant results in these analyses. In summary, the significant region was almost the same as that identified in our initial analyses, although the size of the cluster was reduced. Thus, we used only the region detected in the initial analyses in the following analyses.

**Figure 2:**
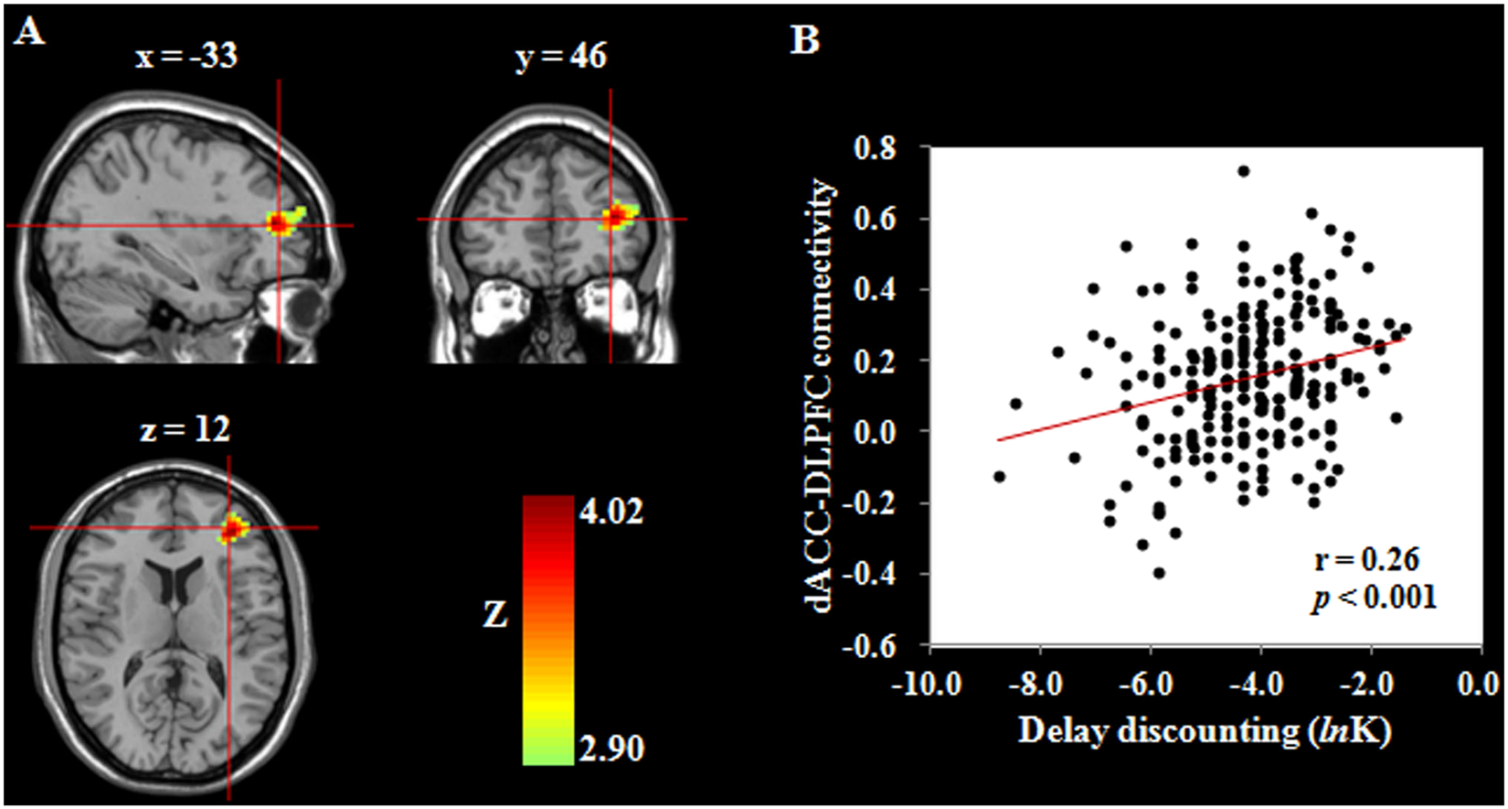
Functional connectivity related to delay discounting. (**A**) Brain images depicting the positive association between delay discounting and the connectivity of the dACC and the left DLPFC. (**B**) Scatter plot showing the correlation between delay discounting and the strength of dACC-DLPFC connectivity (r = 0.26, *p* = 0.00008). dACC, dorsal anterior cingulate cortex; DLPFC, dorsolateral prefrontal cortex.

Second, to further explore the role of dACC in DD, we carried out a seed-based RSFC analysis by using the dACC with significant association with DD as seed ROI and studying its connection to the rest of the brain. Then, we performed whole-brain correlation analyses between the RSFC and DD scores with gender, age and FD as controlling variables. The results revealed that after correcting for multiple comparisons (Monte Carlo simulation), DD was positively related to RSFC strength between the dACC and left DLPFC (the middle frontal gyrus; r = 0.26, *p* = 0.00008; see Fig. 2 and Table 2). No other significant results were obtained in these analyses. Then, to assess the stability of the association between DD and RSFC, we performed prediction analyses using the linear regression and four-fold balanced cross-validation procedure. The strength of the dACC-DLPFC connectivity significantly predicted individual differences in DD [r_(predicted, observed)_ = 0.22, *p* = 0.0007], even after adjusting for gender, age and FD.

### The DD-specific nature of the findings

To test the specificity of the associations between DD and intrinsic brain activity, we excluded the confounding factors of general intelligence and trait impulsivity. DD was negatively correlated with general intelligence [r = -0.14, *p* = 0.042 (uncorrected), *p* = 0.294 (Bonferroni corrected)] and positively correlated with trait impulsivity [r = 0.19, *p* = 0.005 (uncorrected), *p* = 0.035 (Bonferroni corrected)]. Then, we conducted correlation analyses to examine whether general intelligence and trait impulsivity can affect the relationships between DD and fALFF and RSFC. After controlling for general intelligence and trait impulsivity, DD was still related to the fALFF of the dACC [r = 0.28, *p* = 0.00002 (uncorrected), *p* = 0.0001 (Bonferroni corrected)] and the strength of the dACC-DLPFC connectivity [r = 0.26, *p* = 0.00009 (uncorrected), *p* = 0.0007 (Bonferroni corrected)], suggesting that the observed associations were specific to DD. Gender, age and FD were controlled for in these analyses.

Next, we performed hierarchical regression analyses to evaluate how much of the variance in DD can be explained by intrinsic brain activity. The results showed that the fALFF in the dACC [β = 0.29, *p* = 0.000003 (uncorrected), *p* = 0.00002 (Bonferroni corrected)] and the dACC-DLPFC connectivity [β = 0.26, *p* = 0.00003 (uncorrected), *p* = 0.0002 (Bonferroni corrected)] jointly accounted for 14.6% of the variance in DD (*ΔR* ^2^ = 0.146) beyond the variance explained by general intelligence and trait impulsivity as well as gender, age and FD. These results indicated that the fALFF in the dACC and the dACC-DLPFC connectivity uniquely predict individual differences in DD.

## Discussion

In the current study, we sought to examine the functional brain correlates of DD in late adolescents by performing RS-fMRI. At the regional level, we found an association between higher DD and greater fALFF in the dACC. At the connectivity level, higher DD was related to stronger RSFC between the dACC and the left DLPFC. Furthermore, the fALFF in the dACC and dACC-DLPFC connectivity uniquely predicted individual differences in DD. These results persisted even after adjusting for the influences of general intelligence and trait impulsivity, indicating the DD-specific nature of the findings. In brief, the present study reveals that the regional fALFF and RSFC serve as the functional neural basis of DD in late adolescents, which aids to strengthen and corroborate our understanding of the neural underpinnings of individual differences in DD.

Confirming our first hypothesis, the fALFF in the dACC predicted individual differences in DD. This result fits well with those of previous fMRI studies revealing brain activity in the dACC during completing DD tasks^38, 53–56^. The activity of the dACC might reflect its function in conflict monitoring and strategy adaptation, which are considered key mechanisms for biasing future behaviors toward more efficient modes^14, 57^. Moreover, evidence from two structural MRI studies has demonstrated that structural variations in the dACC play a critical role in individual differences in DD^17, 18^. Furthermore, the result of the association between higher DD (worse self-regulation) and greater fALFF is consistent with a magnetic resonance spectroscopy (MRS) study that revealed a negative association between patient behaviors and dACC glutamate concentrations at the resting state^36^. Our results were also consistent with a body of studies reporting higher (f)ALFF in the dACC among patients with self-regulation-related disorders such as obsessive-compulsive disorder^58, 59^, schizophrenia^60^, depressive disorder^61, 62^, and post-traumatic stress disorder^63^. Higher fALFF values in the dACC among impatient participants and patients with self-regulation disorders might reflect an enhanced cortical modulation of neural activities^64, 65^ or a compensatory mechanism to overcome defects in brain function and structure^63, 66^.

Confirming our second hypothesis, our study revealed an association between dACC-DLPFC connectivity and individual differences in DD, which was in line with prior functional and structural findings on DD in the brain. On one hand, DLPFC activities during DD tasks were repeatedly reported in previous studies, which demonstrated the role of DLPFC in exerting self-control to obtain greater long-term benefits^38, 55, 56, 67–69^. On the other hand, the gray matter structure of the DLPFC has been found to be linked with individual differences in DD, supporting the DLPFC as a neuroanatomical marker for DD^16, 18, 19^. In addition, using repetitive transcranial magnetic stimulation (rTMS), Figner *et al*. (2010) reported that transient disruption of the left DLPFC caused participants to select more immediate rewards, providing direct evidence for the causal role of the left DLPFC in DD^70^. Considering that the DLPFC and the dACC are generally considered the core brain regions in human self-regulation system^1, 2^, our finding regarding the association between DD and dACC-DLPFC connectivity supports the role of the cognitive control network in DD to a certain degree^14, 15^.

Several limitations of this study deserve consideration in future research. First, we measured DD by using the Kirby questionnaire, which is a relatively antiquated and crude measure of DD since it only produces measures in certain bands of discounting. Future studies are encouraged to use other dynamic adjusting procedures to measure DD^38, 71^, which may improve the reliability and validity of the measurement. In addition, the monetary reward for MCQ used in this study was hypothetical, although some studies have showed that the discount rates obtained using hypothetical choices are not substantially different from those obtained using real-monetary choices^72–75^. Future investigations could consider using real-monetary reward to measure DD and explore its relations to fALFF and RSFC. Second, the participants of the current study included a group of healthy high school students with a narrow age range, which may limit the generalizability of the findings, although it has the advantage of obtaining sufficient statistical power for the whole-brain analyses. Future studies are necessary to extend our study to include more diverse populations, such as the elderly, adults, children and individuals with psychiatric illnesses. Third, only fALFF and RSFC were used as measures of brain function to examine the neurobiological basis of DD during the resting state. Future studies could consider examining this issue by utilizing other measures of brain function (e.g., task-based brain activity) and structure (e.g., cortical gray matter volume or cortical thickness) and then compare the results across the different brain measures. Finally, the r values of the correlations between DD and general intelligence and trait impulsivity were rather low, which may represent the spurious correlations in considering of the large sample size. These low correlations may caused by the self-report measures used in the current study. For example, evidence from previous studies using self-reported measures has suggested that the relationship between DD behavior and impulsivity is not yet entirely clear^6^. Thus, future investigations are encouraged to use more reliable and valid measures (e.g., behavioral instead of self-reported) to examine the associations between DD, general intelligence and impulsivity.

In conclusion, the present study used RS-fMRI to explore the functional brain substrates of DD in late adolescents. The whole-brain correlation analyses suggested that individual differences in DD were positively related to the fALFF in the dACC and the RSFC between the dACC and left DLPFC. These results remained significant even after adjusting for the effects of general intelligence and trait impulsivity, demonstrating the stable and specific characteristics of our findings. In short, our study provides the evidence of the functional neural basis of DD in late adolescents. Finally, this study might have educational implications, as we provide potential neurobiological markers that can be used by education experts to develop corresponding intervening programs to promote effective decision-making and well-being in adolescents. Moreover, our study may add to Psychoradiology (https://radiopaedia.org/articles/psychoradiology), which is an emerging subspecialty of radiology with growing intersection between the fields of clinical imaging and psychiatry/psychology^76, 77^.

## Methods

### Participants

The participants included 234 right-handed and healthy adolescent students (mean age = 18.60 ± 0.78 years, 122 females), who were part of a longitudinal project that aimed to explore the determinants of social cognition, academic success and well-being among adolescents in Chengdu, China^31, 78–80^. Each of the students had recently graduated (by June 2015) from one of several local public high schools and did not have a history of psychiatric or neurological illness. The experiments were carried out between June 2015 and September 2015; and the Edinburgh Handedness Inventory^81^ was used to measure handedness. Six participants were excluded due to abnormal brain structure (3) or a lack of behavioral test scores (3). Thus, 228 participants (mean age = 18.48 ± 0.55 years, 119 females) were included in the data analyses. The current study was approved by the local research ethics committee of West China Hospital of Sichuan University. Written informed consent was obtained from each participant prior to experimentation. After completing all the measurements, each participant received ¥100 for compensation. The study protocols were performed in accordance with the approved guidelines and regulations.

### Behavioral measures

#### Monetary Choice Questionnaire (MCQ)

We used the 27-item MCQ to measure individual differences in DD^4^. According to the delayed reward magnitudes, the 27 items were grouped into 3 conditions: large (¥75–85¥), medium (¥50–60¥) and small (¥25–35¥), with 9 items per condition. The delay time ranged from 7 days to 186 days. For each item, the participants were asked to choose either a larger, delayed reward or a smaller, immediate reward. For instance, “Would you prefer ¥75 in 20 days or ¥41 today?” Previous evidence has shown that a hyperbolic function fits well with the responses of the participants: V = A/(1 + kD), where A refers to the delayed reward, V refers to the present reward, D refers to the delay time, and k refers to the discount rate parameter^82^. Based on a procedure developed in previous studies^3, 4^, we computed the scores for DD utilizing the following steps. First, we obtained the k value for a given delayed reward condition according to the highest consistency among a participant’s choices. Second, we calculated the geometric mean of the k values of the three delayed reward conditions and then obtained a single k value for each participant. Higher k values represented higher impulsivity (i.e., more likely to select the immediate reward). Finally, we used a natural log transformation to normalize the k values (*lnk*) because the raw k values were not normally distributed. Evidence from previous studies has indicated that the MCQ shows good reliability and validity among adolescents and adults^4, 21, 83^. The MCQ has also been widely used in Chinese populations^84, 85^. To evaluate the internal reliability of the MCQ, the consistency value for each participant (i.e., the percent of the consistent responses) was first calculated and then the average consistency value for all participants was obtained. This average consistency value has been employed in previous investigations^83^. In our dataset, the average consistency values were as follows: for the large delayed reward condition, mean = 98.64%, standard deviation (SD) = 3.65%, minimum = 88.89%, maximum = 100%; for the medium delayed reward condition, mean = 98.73%, SD = 3.69%, minimum = 77.78%, maximum = 100%; for the small delayed reward condition, mean = 98.73%, SD = 3.54%, minimum = 88.89%, maximum = 100%. These high consistency values suggested that the participants made their responses very carefully during testing. Because participants were compensated irrespective of their choices on the questionnaire, we used hypothetical monetary reward in this study.

#### Raven’s Advanced Progressive Matrix (RAPM)

Because general intelligence is found to be associated with DD^11, 86^ and intrinsic brain activity^87^, we employed RAPM^88^ to rule out the possible influences of general intelligence on the associations between DD and intrinsic brain activity. RAPM is one of the most popular and sound instruments for assessing general intelligence and includes 36 non-verbal graphical matrices. During testing, the participants were instructed to identify the missing parts for all items within 30 minutes^89^. The RAPM score was defined as the number of correct answers; the higher the score, the higher the level of general intelligence. In our dataset, the Cronbach’s α value of RAPM was 0.82, suggesting an adequate internal consistency.

#### Barratt Impulsivity Scale-11 (BIS-11)

Considering the associations between trait impulsivity and DD^4, 86^ and intrinsic brain activity^90^, we used the 30-item BIS-11^91^ to exclude the possible effects of trait impulsivity on the associations between DD and intrinsic brain activity. The BIS-11 is a widely used self-report questionnaire for assessing trait impulsivity. The scale includes three subscales: non-planning impulsivity, attentional impulsivity and motor impulsivity. The response option for items ranges from 1 (rarely/never) to 4 (almost always/always). The total score for BIS-11 was computed by summing the scores across all of the items, with higher scores reflecting greater impulsivity. A systematic review of the BIS-11 has reported that the scale exhibits strong reliability and validity across samples from different countries^92^. The Chinese versions of BIS-11 have demonstrated satisfactory psychometric properties among Chinese high school students^93, 94^. In our dataset, Cronbach’s α value of BIS-11 was 0.79, showing an adequate internal consistency.

### Imaging data acquisition and analyses

#### Data acquisition

All participants were scanned using a Siemens-Trio Erlangen MRI scanner (3.0 T, Germany) at the West China Hospital of Sichuan University, Chengdu, China. The scanner was equipped with a 12-channel head coil. First, using a magnetization prepared gradient echo sequence, T1-weighted anatomical images were obtained using the following parameters: TR/TI/TE, 1900/900/2.26 ms; flip angle, 9°; matrix, 256 × 256; voxel size, 1 × 1 × 1 mm^3^; 176 slices. Then, using an echo-planar imaging (EPI) sequence, RS-fMRI data were acquired using the following parameters: TR/TE = 2000/30 ms; flip angle, 90°; field of view, 240 mm × 240 mm; matrix, 64 × 64; slice thickness, 5 mm; inter-slice gap, 0 mm; 30 slices; 240 volumes; voxel size, 3.75 × 3.75 × 5 mm^3^. During scanning, each participant was asked to lie still, remain awake with eyes closed, and think of nothing purposively. Foam pads and earplugs were used to abate head motion and noise perception.

#### Data preprocessing

Prior to preprocessing, a medical radiologist who was blind to this research visually inspected the image data for each participant. Three participants were excluded because of abnormal brain structure (e.g., unusual cyst). To ensure the stability of the MRI signals during adaption in participants, we discarded the first 10 images. Then, the remaining data were preprocessed using the following steps: slice timing and head motion correction, realignment, normalizing with 3 × 3 × 3 mm^3^ resolution, smoothing using an 8 mm FWHM Gaussian kernel and removing linear trends. To remove the influences of nuisance covariates in the fALFF and RSFC analyses, we regressed out six head motion parameters^95, 96^, the global mean signal, the white matter signal, and the cerebrospinal fluid signal. Finally, the data were filtered with a temporal band-pass filter (0.01 - 0.08 Hz, for RSFC except for fALFF)^97^. None of the data were excluded during preprocessing for two reasons. First, the translational and rotational parameters for all of the participants did not exceed ± 1.5 mm and ± 1.5°, respectively. Second, the FD values of participants did not exceed 0.30 and these values were treated as a covariate in the brain-behavior correlation analyses. The preprocessing was performed using SPM8 (http://www.fil.ion.ucl.ac.uk/spm/software/SPM8), which was employed using the DPARSF toolbox^98^.

#### fALFF calculation

We calculated the fALFF based on the procedure developed by previous studies^27, 28^. First, to obtain the power spectrum, we transformed the time courses of each voxel into the frequency domain. Then, after computing the square root of each frequency in the power spectrum, we obtained the mean square root across a low-frequency range (0.01–0.08 Hz). This mean square root is regarded as the ALFF index^99^. Considering that fALFF is a normalized score of ALFF, we calculated the fALFF as a fraction of the sum of the amplitudes across the entire frequency range (0–0.25 Hz). Finally, by subtracting the global mean fALFF and dividing by the standard deviation, we transformed the fALFF map into the fALFF Z-score map for each participant. These analyses were conducted using the DPARSF toolbox^98^.

### Statistical analyses

#### fALFF-behavior correlation analyses

To identify brain regions with spontaneous activity associated with DD, we conducted whole-brain correlation analyses between the MCQ scores and the fALFF values in each voxel, with age, gender and FD as nuisance covariates. We corrected for multiple comparisons using the AlphaSim program in the REST software package^100^, which employs Monte Carlo simulation^101^. Specifically, the threshold for significant clusters was set as follows: 10,000 iterations, *p < *0.05 at the cluster level combined with a *p < *0.005 at the voxel level, at least 50 voxels (1350 mm^3^). AlphaSim is a popular program used in previous studies to analyze RS-MRI data^29–31, 35^.

#### RSFC-behavior correlation analyses

To investigate the relationship of DD and RSFC between the clusters identified from the fALFF-behavior correlation analyses and other clusters across the brain, we carried out RSFC-behavior correlation analyses using REST software^100^. First, we used the clusters with significant associations with DD to create the seed ROIs. Second, we extracted the mean time series from voxels in each seed ROI in each participant. Third, to obtain the participant-level correlation maps, we correlated the mean time series in each seed ROI with that of other voxels across the brain. Fourth, using Fisher’s r-to-z transformation, we converted the correlation maps to Z-score maps. For the group-level analyses, we conducted correlation analyses between the Z-score maps and MCQ scores to detect the RSFC related to DD, with age, gender and FD as nuisance covariates. To correct for multiple comparisons, we applied the AlphaSim program and set the threshold for significant clusters as follows: 10,000 iterations, *p < *0.05 at the cluster level combined with a *p < *0.005 at the voxel level, at least 50 voxels (1350 mm^3^).

#### Prediction analyses

We carried out a machine learning approach to test the stability of the association between DD and intrinsic brain activity. This approach is based on balanced cross-validation using linear regression, which has been widely used in previous studies^78, 102–105^. In this analysis, DD served as the dependent variable and either fALFF or RSFC was used as the independent variable. The predictive ability of independent variable on dependent variable was defined as r_(predicted, observed)_, which was evaluated using a four-fold balanced cross-validation procedure. First, we divided the data by four to ensure that the independent variable and dependent variable distributions across the four divisions were balanced. Second, we used three divisions to build a linear regression model, leaving out the fourth division. Then, we employed this model to predict the data for the fourth division. We repeated this procedure four times to obtain a final r_(predicted, observed)_, which represented the association between the observed data and the data predicted by the regression model. Here, we applied a nonparametric testing method to determine the statistical significance of the model. Specifically, The empirical null distribution of r_(predicted, observed)_ was estimated by generating 1,000 surrogate datasets under the null hypothesis that there was no association between DD and intrinsic brain activity. We generated each surrogate dataset Di of size equal to the observed dataset by permuting the labels on the observed data points^78, 102–105^. Next, we used the predicted labels with the four-fold balanced cross-validation procedure and the actual D_i_ labels to calculate the r_(predicted, observed)_ of D_i_ (i.e., r_(predicted, observed)i_). Finally, we counted the number of r_(predicted, observed)i_ values that were greater than r_(predicted, observed)_ and divided that count by the number of D_i_ datasets (1000). The resulting value was considered the level of statistical significance (p-value). Age, gender and FD were controlled for in these analyses.

#### Hierarchical regression analyses

To examine whether the intrinsic brain activity can explain additional variance when predicting DD beyond other predictors (i.e., general intelligence and trait impulsivity) and demographic factors (i.e., age and gender), we carried out a hierarchical regression analysis using SPSS software (version 22.0). In this analysis, the dependent variable was DD; the independent variables in step 1 were general intelligence, trait impulsivity, age, gender and FD; and the independent variables in step 2 were the fALFF of brain region(s) and the RSFC identified in the prior whole-brain analyses.
